# Trends in distribution of harm-reduction equipment for persons who use drugs in Norway, 2016–2022. Archival analysis based on nationwide data collections

**DOI:** 10.1186/s12954-025-01245-5

**Published:** 2025-05-30

**Authors:** Jens Christoffer Skogen, Katharina Natalie Gottschlich, Martin Blindheim, Janne Årstad

**Affiliations:** 1 Center for Alcohol and Drug Research (KORFOR), Stavanger University Hospital, Stavanger, Norway; 2 Department of Health Promotion, Norwegian Institute of Public Health, Bergen, Norway; 3 Centre for Evaluation of Public Health Measures, Norwegian Institute of Public Health, Oslo, Norway; 4 Research Department, Stavanger University Hospital, Stavanger, Norway; 5 Department of Mental Health and Substance Use, Norwegian Directorate of Health, Oslo, Norway

## Abstract

Harm-reduction strategies are interventions designed to mitigate the adverse effects of substance use, without requiring abstinence. Evidence supports the effectiveness and efficacy of harm reduction as a broad framework for addressing illicit drug use. To ensure the implementation of these measures, Center for Alcohol and Drug Research (KORFOR) was mandated by the Norwegian Directorate of Health in 2016 to annually assess municipalities’ adherence to harm-reduction guidelines. This study aims to present national trends in the distribution of harm-reduction equipment for the prevention of infectious diseases, specifically needles and syringes and smoking foil. We investigated the proportion of distributing municipalities, additional equipment distributed (naloxone, condoms and lubricants, disposable toothbrushes, and cookers, filters, disinfection swabs, sterile water, and ascorbic acid), and the population coverage. Our findings indicate an increased coverage in the distribution of harm-reduction equipment in Norway between 2016 and 2022. This positive trend demonstrates progress in addressing the negative consequences of drug use and aligns with Norwegian national strategies to mitigate drug-related harms. Future research should evaluate the effectiveness of these harm-reduction strategies and identify areas for improvement within the Norwegian context, especially related to use of opioid analgesics.

## Introduction

Harm-reduction strategies can be described as a set of interventions which aim to reduce the negative consequences associated with drug use, without requiring abstinence [1, 2]. As a strategy it can be applied to alcohol, tobacco, illicit drugs, and other harmful health behaviors [3]. However, they are most associated with illicit drugs [1], particularly opioids and stimulants [4]. The specific interventions employed in harm-reduction strategies are varied, and cover for example peer education, health promotion, opioid substitution treatment and needle and syringe programmes [4]. Evidence supports the effectiveness and efficacy of harm-reduction as a broad framework for addressing illicit drug use [1, 4]. Current opioid agonist treatment (OAT) was reported to be associated with substantially reduced health risks and lower levels of criminal activity compared to other groups (former OAT users and those never in OAT) in a population of needle exchange programme participants in a Norwegian context [5].

Building upon harm-reduction strategies, the availability and accessibility of needles and syringes for people who inject drugs in Norway have undergone substantial changes over the past five decades, shaped by public health crises and evolving strategies to address risks associated with drug injection. Historically, needles and syringes were available over-the-counter in pharmacies [6], but their sale was subject to various local restrictions targeting persons who inject drugs. During the height of the hepatitis B (HBV) epidemic in the late 1970s, many of these restrictions were lifted (Torbjørn Mork, personal communication). The landscape shifted again in 1985, with the AIDS epidemic among persons who inject drugs. Fearing HIV transmission, some Norwegian pharmacies stopped selling needles and syringes to avoid serving persons who inject drugs as customers. During the summer of 1985, activists took matters into their own hands by distributing needles and syringes in the streets (Martin Blindheim, personal communication). Several municipalities started experimenting with various distributing methods [6] and successfully put pressure on the pharmacies to stop this discriminatory practice.

In 1988, the AIDS-information bus was launched in the capital (Oslo), Norway’s first permanent needles and syringes program [7, 8]. This bus operated six hours daily, 365 days a year. In 1989, it distributed 212,000 needles and syringes, and the number surged to 1,136,000 in 1995; nearly as many as today despite fewer persons who inject drugs. In the 1990s, the Norwegian government started funding low-threshold health facilities in major cities, with needle and syringes distribution becoming central in harm reduction efforts. Oslo opened a drug injection room in 2005, followed by Bergen (the second largest city in Norway) in 2016, providing a safer environment for drug use while aiming to reduce associated risks [9].

In 2015 the National Overdose Strategy and in 2016 the Hepatitis Strategy emphasized the need for increased availability of needles and syringes [10, 11], aligning with the World Health Organization’s (WHO) Hepatitis Strategy [12]. The WHO strategy guides the health sector in implementing focused responses to end AIDS, viral hepatitis B and C, and sexually transmitted infections by 2030. Among the targets to achieve this, is a distribution of at least 300 needles and syringes for persons who inject drugs [13]. To ensure the implementation of harm reduction measures in Norway, Center for Alcohol and Drug Research (KORFOR) was mandated in 2016 to annually assess the municipalities’ adherence to availability recommendations and other harm-reduction measures.

By 2018, the Directorate of Health required by law that municipalities where people who use drugs live or stay provide free sterile injecting equipment and smoking foil, linking this mandate to the Act on Control of Communicable Diseases [14] and the ongoing HCV-epidemic [15]. Thus, the distribution of such harm-reducing equipment was stipulated for the prevention of infectious diseases. The mandate indirectly related to overdose prevention efforts by assuming that infections will make a person more susceptible to overdose. It is worth stressing that in the Norwegian context only the distribution of needles and syringes, and smoking foil is a legal obligation for the municipalities, whereas additional equipment (naloxone, contraception and lubricants, cookers, filters, disinfection swabs, sterile water, and ascorbic acid) is merely recommended. The recommended equipment is related to prevention of infectious diseases and for naloxone it is part of the Norwegian national overdose strategy. It is also worth noting that sterile needles and syringes have always been distributed together as a kit in Norway, and never separately [8].

In 2019, drug injection rooms were reorganized as “user rooms”, promoting less harmful drug use methods. The user rooms allowed for a broader range of harm reduction strategies, not limited to injecting alone [9]. This shift represented a significant change in supporting persons who inject drugs and addressing public health concerns associated with drug use. Only Oslo and Bergen (the two largest cities in Norway) have so far opened user rooms. However, most distribution is done from other facilities, also in these two cities.

In 2024, the largest municipality by population in Norway is Oslo with ~ 560 000 inhabitants aged 18 or more, while the smallest by population is Utsira with 176 inhabitants as of 2024 [16]. There are 48 municipalities with a population size of at least 20 000 aged 18 or more.

The present paper addresses aspects of the Norwegian harm-reduction strategies by presenting nationwide pertinent data on the distribution of harm-reduction equipment in Norway offering insights into the reach, accessibility, and inclusivity of the harm reduction initiatives across Norway.

## Aims

The aim of this study is to present the national trends in the distribution of harm-reduction equipment in Norway from 2016 to 2022 aimed at the prevention of infectious diseases, specifically needles and syringes and smoking foil. We investigated the proportion of distributing municipalities and the population coverage. Additionally, we investigated the distribution of naloxone, condoms and lubricants, disposable toothbrushes, and other additional equipment.

## Methods

### Data collection and procedure of municipality surveys regarding distribution

On behalf of the Norwegian Directorate of Health, Center for Alcohol and Drug Research (KORFOR), has conducted a yearly electronic questionnaire survey to all Norwegian municipalities regarding the distribution of harm reduction equipment in the period between 2016 to 2022. Harm reduction equipment in this context refers to equipment used for the consumption of substances (needles and syringes and smoking foil), as well as naloxone, condoms and lubricants, disposable toothbrushes, the aggregate category “cookers, filters, alcohol swabs, ascorbic acid and/or sterile water”. The questionnaire was developed based on input from “Funkishuset”, a low-threshold initiative in Sandnes Municipality and their users, and input from the Norwegian Directorate of Health. Emphasizing a high response rate, the questionnaire was designed to be completed within 10–15 minutes. The survey was electronic (online), and was sent to the municipality’s post office, with a request to forward it to the chief medical officer, who is responsible for the prevention of infectious diseases in the municipalities. The chief medical officer was requested to forward the survey to the person most knowledgeable in the area if they themselves were not the most informed. During the next 3–4 months, three reminders were carried out. The first reminder was sent to the municipality’s post office, the second was sent to available contact persons in each municipality and the third reminder was carried out by telephone to municipalities of a certain size (≥ 100 000 inhabitants). For each survey year, the Center for Alcohol and Drug research (KORFOR) published a report based on results from the data collected.

### Data retrieval

The approach used in the present study is secondary analysis of existing data (archival data). The data used in the present study is derived from three sources. Data about distribution is derived from the official yearly reports and underlying data issued between 2017 and 2023 by Center for Alcohol and Drug research (KORFOR) commissioned by the Norwegian Directorate of Health (data source 1; [17]). The retrieved information from these reports includes number of participating municipalities, number of distributing municipalities, and estimated needles and syringes distributed as well as the number of municipalities distributing other equipment: (i) smoking foil, (ii) naloxone, (iii) condoms and lubricants and (iv) disposable toothbrushes and (v) cookers, filters, alcohol swabs, ascorbic acid and/or sterile water. The latter was an aggregate category. The questions regarding distribution of (i-iv) are only available from 2017 and onwards and the questions about the distribution of (v) are only available from 2019, as these specific questions were not included in the questionnaire from the beginning. The main outcomes in the present study were distributing municipalities and distribution of needles and syringes and smoking foil, and distribution of additional equipment as listed above (ii-v).

The total number of municipalities and population size are retrieved from official governmental information (data source 2; [18]) and Statistics Norway (data source 3; [19]), respectively.

### Statistical procedure

Table 1 shows an overview of all included variables for each year between 2016 and 2022. Total numbers are given for each variable at an aggregate level. For the estimated proportions, the denominator is the total number of municipalities each year. For the total population covered, the proportion is estimated with the total population as the denominator. Tests for trends in distribution were done using the Cochran-Armitage test for trend of proportions, with total municipalities for each year as the denominator. All analyses were done using Stata/SE 18 for Windows [20].

## Results

The proportion of participating municipalities was 59% in 2016, increasing to between 70% and 73% from 2017 to 2019. During the peak of the COVID-19 pandemic (2020–2021), the participation dropped to 62–64%, but rose again to 70% in 2022 (Table 1). Throughout the period, the proportion of distributing municipalities increased, from 16% in 2016 to 43% in 2022, an increase of 27% points (*p* < 0.001; Table 1). There was also an increase in the distribution of all equipment apart from the aggregate category “cookers, filters, alcohol swabs, ascorbic acid and/or sterile water” (*p* = 0.255). For distribution of needles and syringes, the proportion increased from 11% in 2016 to 36% in 2022 (an increase of 26% points, *p* < 0.001), while the comparable proportion for smoking foil was 7% and 16% (an increase of 9% points from 2017 to 2022, *p* < 0.001). The estimated population covered also increased from 52% in 2016 to 80% in 2022 (an increase of 28%, *p* < 0.001). For all significant trend tests, there was no indication of a departure from trend (all p-values > 0.05), which may be taken as support for linear trend. The total number of estimated needles and syringes distributed also increased during the period, although there was a drop from 2021 to 2022.

**Table 1:** Overview of included variables across years 2016–2022, and tests for trends in distribution

	2016	2017	2018	2019	2020	2021	2022	Test for trend^c^	Percentage points difference^d^	
Municipalities	428	426	422	422	356	356	356	-	-	
Participating municipalities (% of total municipalities)	253 (59%)	298 (70%)	306 (73%)	300 (71%)	227 (64%)	220 (62%)	246 (69%)	-	-	
Distributing municipalities (% of total municipalities)	67 (16%)	93 (22%)	111 (26%)	148 (35%)	135 (38%)	132 (37%)	154 (43%)	*p* < 0.001	+ 27	
Municipalities distributing:										
Needles and syringes (% of total municipalities)	45 (11%)	67 (16%)	90 (21%)	124 (29%)	117 (33%)	116 (33%)	128 (36%)	*p* < 0.001	+ 26	
Smoking foil (% of total municipalities)	*Not included*	29 (7%)	39 (9%)	58 (14%)	57 (16%)	51 (14%)	56 (16%)	*p* < 0.001	+ 9	
Naloxone (% of total municipalities)	*Not included*	23 (5%)	34 (8%)	53 (13%)	52 (15%)	61 (17%)	78 (22%)	*p* < 0.001	+ 17	
Condoms and lubricants (% of total municipalities)	*Not included*	68 (16%)	78 (18%)	85 (20%)	74 (21%)	73 (21%)	86 (24%)	*p* = 0.005	+ 8	
Disposable toothbrushes (% of total municipalities)	*Not included*	35 (8%)	44 (10%)	47 (11%)	40 (11%)	39 (11%)	48 (13%)	*p* = 0.033	+ 5	
Cookers, filters, alcohol swabs, ascorbic acid and/or sterile water (% of total municipalities)	*Not included*	*Not included*	*Not included*	93 (22%)	80 (22%)	85 (24%)	90 (25%)	*p* = 0.255	-	
Total population, Norway^a^	5 213 985	5 258 317	5 295 619	5 328 212	5 367 580	5 391 369	5 425 270	-	-	
Population covered^b^	2 700 337 (52%)	3 031 460 (58%)	3 407 447 (64%)	3 804 792 (71%)	4 183 080 (78%)	3 977 428 (74%)	4 340 015 (80%)	*p* < 0.001	+ 28	
Estimated needles and syringes distributed	3 029 344	~ 2 900 000	~ 3 000 000	~ 3 000 000	3 522 411	3 783 134	3 458 614	-	-	

^a^Total population retrieved from Statistics Norway. ^b^Population covered based on the total population in distributing municipalities. Municipality population retrieved from statistics Norway. ^c^Cochran-Armitage test for trend of proportions. ^d^Percentage points difference between first year of observation and 2022 in the case of a significant trend as indicated by the Cochran-Armitage test

## Discussion

### Summary of main findings

In the present study, we investigated the national trend of distribution of harm-reduction equipment through harm reducing services in Norway from 2016 to 2022. Overall, the findings indicated an increase in distribution over the years covered, especially for needle distribution where the number of units distributed were reported. The proportion of distributing municipalities increased by 27% points from 2016 to 2022 and covered 80% of the Norwegian population by 2022. Collectively, the observed increase in coverage may be taken as support for an ability to mitigate the negative consequences associated with substance use in Norway. This observed upward trend in distribution is also in line with previous estimations made by the Norwegian Institute of Public Health (NIPH).This indicates the number of needles and syringes distributed to individuals who inject drugs in Norway increased during the same period (2015–2021), with point estimates going from 286 (CI95% 237–335) in 2015 to 482 (443–566) in 2021 [21]. In the same period, the estimated number of persons injecting drugs in Norway decreased from ~ 8700 in 2015 to ~ 7900 in 2021, with an average of about 8300 across years [21]. The WHO Hepatitis strategy aims at eliminating hepatitis B and C as a major public health threat by 2030. Among the targets that should be met by 2030 is the distribution of at least 300 needles and syringes per person who injects drugs [13]. According to the national estimates provided by NIPH, this target was met in 2016 (point estimate 349). Recent estimates from the European Union Drugs Agency barometer also indicate a decrease in the prevalence of hepatitis C infection in Norway (Oslo) from 2015 (46%) to 2022 (9%) among people who inject drugs [22]. Note that these estimates cover Oslo only. Our findings are also supported by the most recent Global Burden of Disease (GBD) estimates that indicates a statistically significant decrease from 3.6 (95% uncertainty interval (UI): 3.4–3.8) age-standardized deaths per 100,000 in 2016 to 3.1 (95%UI: 2.9–3.4) in 2021 due to opioid use disorders for Norway [23]. In fact, the GBD estimates indicate a consistent downward trend in age-standardized deaths due to opioid use disorders in Norway after a peak in 2001 (6.5 (95%UI 6.2–6.8) and are now more alike the other Nordic countries than at the start of the millennium [24]. Overall, the present findings are indicative of a progress towards achieving the goals outlined in Norwegian national strategies aimed to address substance use and promoting public health. Notably, the trend in drug-induced deaths in Norway has shown a slight increase since 2013 [25]. Although the causes for this increase are complex, a shift from heroin-induced deaths to those induced by other opioids has been observed. A marked increase in deaths induced by synthetic opioids is especially noteworthy. This is indicative of change in the underlying demographic, particularly for deaths associated with opioid analgesics [26], indicating that harm-reduction efforts need to be adjusted to ensure they are effectively reaching and relevant to the target population.

### Strengths and limitations

The present study holds notable strengths. First, the study presents detailed data about trends in distribution across a six-year period at the municipality level in Norway. Second, as the study relies on official yearly reports issued by Center for Alcohol and Drug Research (KORFOR) as appointed by the Norwegian Directorate of Health it lends credibility and reliability to the data. Certain limitations should be considered when interpreting the presented findings. First, although the participation rate at a municipal level increased after 2016, the number of municipalities not participating is relatively high. However, it is worth keeping in mind that most of the municipalities not participating are among the least populous in Norway– while the most populous municipalities are participants across the years. This is especially important to note, as > 80% the Norwegian population lives in the 100 (out of 356) most populous municipalities [27]. Second, as this study is based on retrieved aggregated data and was executed in a period with large reductions in municipalities due to mergers [18], accounting for dependence across years for municipality as a statistical unit is not possible. Therefore, inferential statistics is based on trend tests, even though the statistical units under observation are dependent and in principle could be paired. We believe the results from this approach to still be valid as the participation rate has increased, and almost all the trend indicators increased during the period. For this interpretation to be void, the inflated type I error rate must be very strong and uniform, which we believe to be unlikely. For the same reasons, comparisons across groups of municipalities are also not possible based on the available data. We do, however know from the published reports that among the 65–66 (depending on year of observation) largest municipalities by population size, covering just above 70% of the Norwegian population across the survey years, the participation rate was 75% in 2016 compared to 85% in 2022, with an overall average of 82%. And all of municipalities with more than 100 000 inhabitants in 2024 participated in each survey and reported that they distributed needles and syringes. Third, for the years 2017–2019, the estimated needles distributed were more approximate than for the other years, as some of the municipalities did not report exact numbers but rounded estimates of needles and syringes distributed. We do, however, believe that the reported numbers reflect the actual distribution, and if anything is biased downward when considering the more exact numbers reported before and after this period. Fourth, we do not have information about the number of other equipment distributed, and this limits our ability to investigate the amount distributed of smoking foil, naloxone, condoms, and lubricants, and disposable toothbrushes and so on. Fifth, we do not have information about the population in need for the years covered. As such we are not able to calculate the (un)met need for the target population. However, we have presented national estimates of needles and syringes distributed per individual injecting drugs provided by NIPH for the period between 2015 and 2021, which gives an indication of the met need for the target population in that period.

### Relevance and implications

The distribution of harm reducing equipment to individuals with substance use disorders is generally considered an effective measure for reducing harm associated with drug use [1, 4], such as risk for infectious diseases [28]. Specifically, the ECDC/EMCDDA guidelines, advocate the provision of needles and syringes as part of the key interventions for the prevention and control of infectious diseases among people who inject drugs [29]. Furthermore, the distribution facilitates engagement between users and support services [30, 31]. This engagement may foster a sense of self-worth among users and may reduce stigma [31]. Additionally, the threshold for seeking other interventions or substance use treatment is lowered when a connection with support services is already established through the distribution of harm reduction equipment [32].

The current distribution of needles and syringes, naloxone, and other equipment are all efforts associated with the ongoing hepatitis [33] and overdose strategies in Norway [10, 11]. The strategies have relied on a network of municipalities and dedicated personnel responsible for the implementation at local and regional levels. The concerted effort at multiple levels of governance, ranging from central to local, has been a crucial precondition for achieving more effective coverage in Norway. Moreover, at every stage, user representatives have been actively involved, ensuring that the initiatives have had support at the user level and incorporating their perspectives and needs. The same level of involvement applies to personnel in low-threshold services. Lastly, the concerns regarding potential overdoses and related harm have gathered significant attention at the political level, contributing to a relatively robust and coherent political impetus. For instance, in both the National Overdose Strategy [11] and the Escalation Plan for the Substance Abuse Field [34], the Norwegian government aims to assess the need for expanding access to harm reduction equipment. Although we observe an upward trend, municipalities that do not distribute should and will be encouraged to do so. The accountability for monitoring municipalities that do not engage in distribution is given to the Norwegian Directorate of Health. The Norwegian Directorate of Health has mandated regional governors to ensure that distribution is incorporated into local plans and that measures are implemented accordingly.

## Conclusions

In the present study, we found an increased coverage in distribution of harm-reduction equipment in Norway between 2016 and 2022. The observed positive trend indicates progress in addressing the negative consequences of drug use and aligns with Norwegian national strategies aimed at mitigating the harms associated with drug use. Future research should assess the effectiveness and identify areas for improvement in the harm reducing strategies in a Norwegian context, especially related to use of opioid analgesics.

## Data Availability

The published reports from Center for Alcohol and Drug research (KORFOR) based on the surveys are available online (Brukerplan - Helse Stavanger HF). The data used and analysed during the current study are available from the corresponding author on reasonable request (in Norwegian only), including collated data. In addition, data from official Norwegian sources were employed, and these are available online from Statistics Norway and the Norwegian Government (in Norwegian only).
